# Full-Endoscopic Discectomy in an Adult Postoperative Spinal Dysraphism Patient: A Case Report

**DOI:** 10.7759/cureus.29878

**Published:** 2022-10-03

**Authors:** Takeshi Kaneko, Yuichi Takano

**Affiliations:** 1 Spine Surgery, Inanami Spine and Joint Hospital, Tokyo, JPN

**Keywords:** spine discectomy, discectomy, lumbar disc herniation, spina bifida occulta, myelomeningocele, full endoscopic spine discectomy, tethered cord syndrome

## Abstract

A 52-year-old male patient presented with right lumbar pain and was diagnosed with a herniated disc using MRI. Based on his symptoms, a full-endoscopic discectomy via transforaminal approach (FED-TF) was planned. However, we focused on the possibility of an abnormal spinal anomaly due to a history of prior surgery for spina bifida during childhood that was identified through an interview. Although the FED-TF approach is sometimes used in the upper lumbar spine, this approach may damage the existing nerve in the Kambin’s triangle due to an abnormal nerve root course; therefore, we changed the surgical approach to a full-endoscopic discectomy via interlaminar approach (FED-IL) and successfully completed the surgery. Despite the rarity of spinal dysraphism in children with an estimated incidence of 0.05-0.25 per 1000 live births, the probability of these patients undergoing spinal surgery in adulthood is unknown, and the total percentage of those requiring surgery is likely to be small. However, the possibility of abnormal nerve course should be considered when planning surgery for these patients. Therefore, it is important to pay attention to the route of the root and to obtain a preoperative coronal MRI in patients who have undergone spinal surgery during their childhood. Careful interviews and preoperative imaging are of critical importance for the safety and efficacy of spine surgery.

## Introduction

Nerve distribution in the intervertebral foramen is one of the most important safety considerations in full-endoscopic discectomy via transforaminal approach (FED-TF) [1], given the fact that the transforaminal approach provides access to the intervertebral discs through the foramen over a safety zone known as the “Kambin’s triangle.” Kambin’s triangle is a triangular space that is located over the dorsolateral intervertebral disc of the lumbar spine. Patients with a previous history of spinal dysraphism surgery during their childhood with a low-lying conus on MRI may have preoperative abnormalities in their nerve root trajectory, which could potentially compromise the safety of the approach. In this report, we describe a full-endoscopic discectomy via interlaminar approach (FED-IL) that was performed in place of FED-TF as an emergency measure for a patient with an abnormal nerve root course of the exiting nerve due to a prior spina bifida surgery during his childhood.

## Case presentation

**Figure 1:**
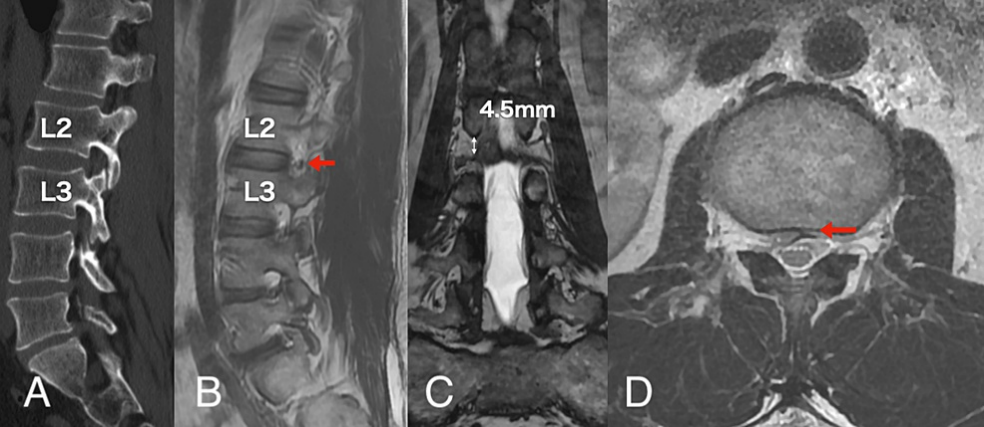
Preoperative imaging (A) CT imaging showed a wide intervertebral foramen at the L23 level. (B) The sagittal MRI showed that the course of the nerve was located at Kambin’s triangle (arrow). (C) The coronal MRI showed that the nerve root was located 4.5 mm distal to the L2 pedicle (arrow). (D) The axial MRI demonstrated that the dura mater was compressed by the herniation that protruded into the spinal canal (arrow).

**Figure 2:**
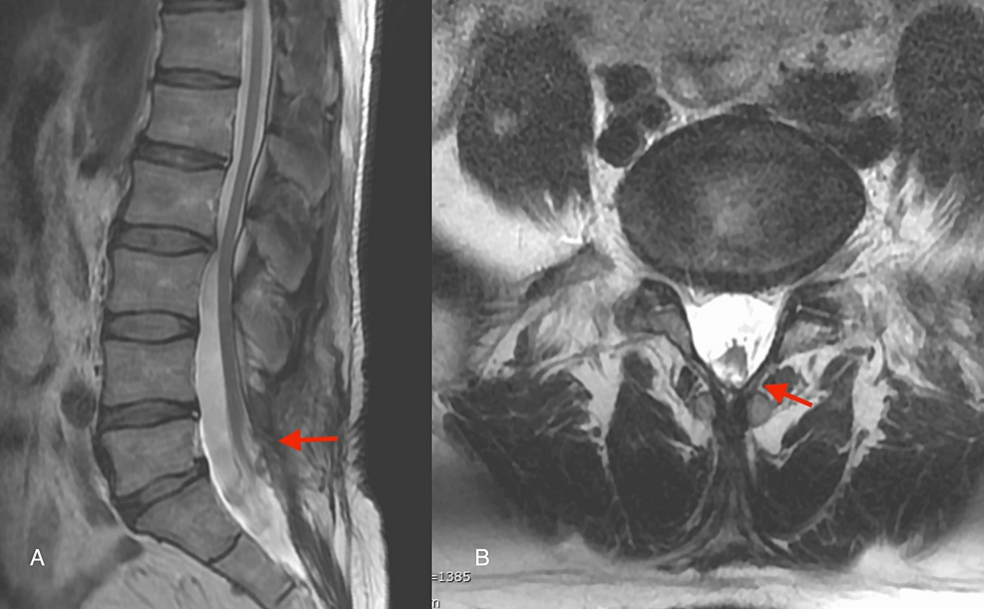
Preoperative imaging (A) The sagittal MRI showed that the descending spinal cord cone was contiguous with a subcutaneous lipoma (arrow). (B) The axial MRI shows a dorsal displacement of the spinal cord (arrow).

**Figure 3:**
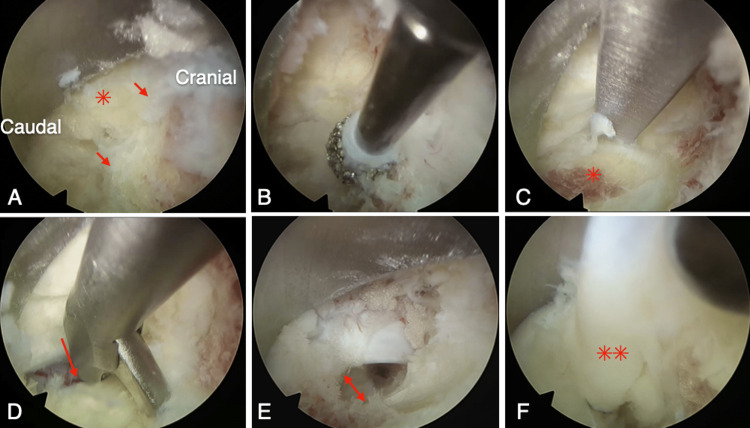
Right intraoperative image at the L23 level (A) Identification of the inferior margin of the lamina (arrow) and the ligamentum flavum (asterisk). (B) Drilling of the inferior margin of the lamina to obtain working space. (C) After drilling the inferior margin of the lamina, the L3 superior articular process is exposed (asterisk). (D) The ligamentum flavum is split near the midline and resected from the midline to the lateral side (arrow). (E) The superior articular process is resected to provide sufficient space on the lateral side of the dural tube (double arrow). (F) The herniation is removed without retracting the dural tube (double asterisk).

**Figure 4:**
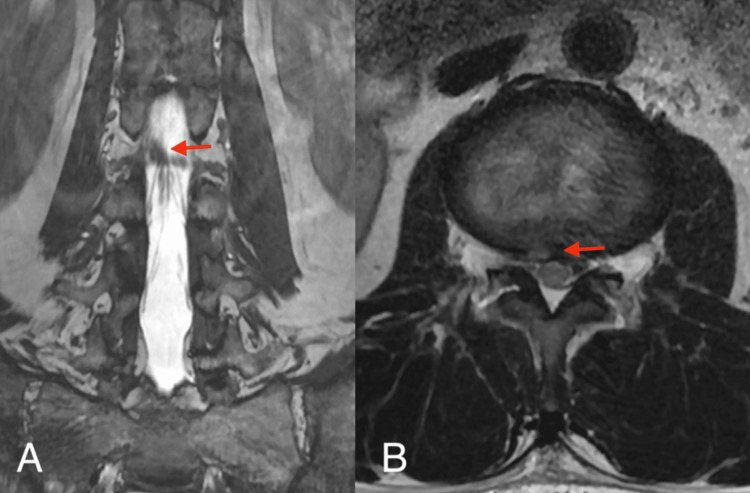
Three-month postoperative MRI (A) Although the position of the exiting nerve root remained unchanged in the coronal MRI, a reduction of upward herniation was observed (arrow). (B) The axial MRI showed the reduction of the herniation that was protruding from the spinal cord (arrow).

A 52-year-old man presented with right low back pain that started several months prior to visiting our outpatient clinic. The patient demonstrated symptoms with acute onset. The pain was persistent and increased during dorsiflexion. During the medical interview, he stated that he had a history of spina bifida closure due to spinal dysraphism when he was one year of age, and there was a surgical scar of approximately 10 cm in length that spanned from the lumbar to the sacral region. He had no history of surgery for hydrocephalus or scoliosis. His symptoms were resistant to medical treatment (mirogabalin 20 mg and tramadol). Three-dimensional computed tomography (3D-CT) demonstrated a large L2/3 foraminal window (Figure 1A). Lumbar magnetic resonance imaging (MRI) revealed an upward-migrated lumbar disc hernia (LDH) at the L2/3 disc level and a compressed L2 nerve root (Figures 1C-1D). Because reproducibility was observed in the L2 nerve root block, the culprit lesion was identified as herniation at the L23 level. Sagittal MRI showed a low-lying medullary cone that was contiguous with a subcutaneous lipoma (Figure 2). The location of the nerve root was within the intervertebral foramen in Kambin’s triangle area (Figure 1B), which made the FED-TF approach difficult. The coronal MRI also showed that the nerve root was 4.5 mm distal to the cranial pedicle (Figure 1C). We determined that the approach via the trans-Kambin area was difficult and adopted FED-IL as an alternative procedure. Under general anesthesia, we performed a FED-IL after insertion to the inferior border of the lamina. Due to the narrow laminar space in the upper lumbar vertebrae, it was difficult to reach the herniation and working channel (Figure 3A). A 3.5-mm high-speed drill (NSK-Nakanishi Japan, Tokyo, Japan) was used to carve out the lateral superior articular process (Figure 3B). Even at the L23 level, the dura mater should not be retracted due to its proximity to the spinal conus. Therefore, the superior articular process was exposed, bone resection was performed with a drill and Kerrison rongeur, and sufficient space was created outside of the dura mater (Figures 3C-3E). A Penfield probe was subsequently used to confirm that the apex of the hernia was reached. The sequestrated nucleus was pulled out without retracting the dural tube (Figure 3F). No drainage was applied. Improved lower back pain was observed at four hours postoperatively. A three-month postoperative MRI demonstrated reduced herniation (Figure 4). The one-year postoperative outcome demonstrated a full recovery (Table 1).

**Table 1:** Preoperative, three-month postoperative, and one-year postoperative outcome of FED-IL for an L2/3 LDH with spinal dysraphism FED-IL: full-endoscopic discectomy via interlaminar approach; ODI: Oswestry Disability Index; EQ5D: EuroQol 5 Dimension; RDQ24: Roland-Morris Disability Questionnaire; JOABPEQ: Japanese Orthopaedic Association Back Pain Evaluation Questionnaire; LDH: lumbar disc hernia.

Assessment	Preoperative	Three-month postoperative	One-year postoperative
ODI	54	2	0
EQ5D	0.77	1	1
RDQ24	15	0	0
JOABPEQ			
Low back pain	0	100	100
Lumbar function	42	100	100
Walking ability	29	100	100
Social life function	73	65	100
Mental health	56	82	91

## Discussion

The incidence of spinal dysraphism ranges from 0.05 to 0.25 per 1000 live births [2], of which the tethered cord syndrome (TCS) is symptomatic in 2-30% of cases [3,4]. In the present case, the low-lying nerve root was also low-lying within the intervertebral foramen along with a low-lying conus. In FED-TF, the intervertebral discs are accessed via Kambin’s triangle [1]. However, Kambin’s triangle cannot be penetrated if a nerve root is present in the safe zone. It is reported that the average distance from the superior pedicle to the nerve root is 1.7 mm [5,6]. In the present case, the deviation from the cranial pedicle to the distal side was 4.5 mm. It is important to observe not only the course of the nerve root with sagittal MRI but also with coronal magnetic resonance myelography. The sagittal MRI showed that the caudally displaced medullary cone was contiguous with a subcutaneous lipoma. In the present case, we were only informed during a medical interview that the patient underwent surgical closure of a spinal defect when he was one year of age. There was no history of surgery for hydrocephalus, paraparesis, or scoliosis. Although differential diagnoses of spina bifida occulta, myelomeningocele, and lipomyelomeningocele were considered, we were unable to achieve a definitive diagnosis. Nevertheless, in addition to the presence of a low-lying conus, surgeons should also consider the abnormal course of nerve roots during the preoperative planning of cases with a history of postoperative spinal dysraphism in childhood. It is important to look at coronal as well as sagittal MRI images to determine the course of the nerve root [7]. In this report, we changed our approach from the FED-TF to the FED-IL approach as an emergency measure. In the present case, the spine was present at the L2/3 level. Because the FED-IL approach involves the retraction of the dura mater to remove herniation, care must be taken in regard to spinal compression [8]. In the present case, it was desirable to drill the L3 superior articular process and secure enough lateral space to remove the herniation. Although no paralysis occurred in this case, the surgical procedure had to be performed while keeping in mind that the surgical site was within unusual proximity to the medullary cone. If the lateral side is shaved excessively, there is a descending exiting nerve; therefore, it is important to understand the positional relationship of the nerves in the coronal MRI. The probability that a patient with a history of spinal dysraphism will undergo spine surgery in adulthood is unknown and is likely to be a small percentage of the total number of surgeries. However, the possibility of abnormal nerve course should be considered when planning surgery for these patients. Therefore, it is important to pay attention to the path of the root and to perform a coronal MRI before surgery in patients who have undergone spinal surgery during childhood.

## Conclusions

It is important to detect abnormalities in the nerve path in advance. Particular attention should be paid to patients with a prior history of spinal surgery during their childhood. Caution should be exercised when a normal anatomical location is not observed. Post-spinal dysraphism cases should be planned with the assumption that low-lying nerve roots may also be present, wherein the coronal MRI is important to determine the appropriate approach for full-endoscopic discectomy.
